# Chronic Venous Disease Patients Showed Altered Expression of IGF-1/PAPP-A/STC-2 Axis in the Vein Wall

**DOI:** 10.1155/2020/6782659

**Published:** 2020-12-14

**Authors:** Miguel A. Ortega, Oscar Fraile-Martínez, Ángel Asúnsolo, Clara Martínez-Vivero, Leonel Pekarek, Santiago Coca, Luis G. Guijarro, Melchor Álvarez-Mon, Julia Buján, Natalio García-Honduvilla, Felipe Sainz

**Affiliations:** ^1^Department of Medicine and Medical Specialities, Faculty of Medicine and Health Sciences, University of Alcalá, Ramón y Cajal Institute of Sanitary Research (IRYCIS), University Center for the Defense of Madrid (CUD-ACD), Alcalá de Henares, 28047 Madrid, Spain; ^2^Department of Surgery, Medical and Social Sciences, Faculty of Medicine and Health Sciences, University of Alcala, Ramón y Cajal Institute of Sanitary Research (IRYCIS), Alcala de Henares, Madrid, Spain; ^3^Department of System Biology, Unit of Biochemistry and Molecular Biology (CIBEREHD), University of Alcala, Alcalá de Henares, Spain; ^4^Immune System Diseases-Rheumatology, Oncology Service and Internal Medicine, University Hospital Príncipe de Asturias (CIBEREHD), Alcalá de Henares, Madrid, Spain; ^5^Angiology and Vascular Surgery Service, Central University Hospital of Defense-UAH, Madrid, Spain

## Abstract

Chronic venous disease (CVeD) has a remarkable prevalence, with an estimated annual incidence of 2%. It has been demonstrated how the loss of homeostatic mechanisms in the vein wall can take part in the physiopathology of CVeD. In this regard, it has been described how different axis, such as IGF-1/PAPP-A/STC-2 axis, may play an essential role in tissue homeostasis. The aim of this research is to study both genetic and protein expressions of the IGF-1/PAPP-A/STC-2 axis in CVeD patients. It is a cross-sectional study in which genetic (RT-qPCR) and protein (immunohistochemistry) expression analysis techniques were accomplished in saphenous veins from CVeD patients (*n* = 35) in comparison to individuals without vascular pathology (HV). Results show a significant increase in both genetic and protein expressions of PAPP-A and IGF-1, and a decrement STC-2 expression at the same time in CVeD patients. Our study is a pioneer for demonstrating that the expression of the different components of the IGF-1/PAPP-A/STC-2 axis is altered in CVeD patients. This fact can be a part of the loss of homeostatic mechanisms of the venous tissue. Further research should be destined to deepen into alterations of this axis, as well as to evaluate the usage of these components as therapeutic targets for CVeD.

## 1. Introduction

Chronic venous disease (CVeD) is described as a complex of disorders that affect the venous system, in which the presence of varicose veins (VV) implies the most important clinical manifestation [1]. It is a condition of great prevalence, more common amongst women and people aged 40-80 years, with an estimated annual incidence of 2% [2]. Amongst the risk factors associated with chronic venous disease, family history, aging, obesity, sedentarism, pregnancy, hormonal factors, and greater height are of great significance [3, 4]. Furthermore, CVeD implies a great cost to the health system, especially in occidental societies [3]; consequently, it is necessary to keep expanding the knowledge of this disease towards a more appropriate management.

Usually, CVeD affects lower limbs, producing venous hypertension which the difficulties of the venous blood return to the heart, having been described as the valvular failure as a key factor of this process [5, 6]. Several authors propose that vein wall alterations can precede those processes. The mechanisms, described in regard to varicose veins, are of great importance: remodelling of the extracellular matrix [7], cellular hypoxia mechanisms [8], and multiple hemodynamic changes which, amongst other effects, conduce to secondary inflammation and oxidative stress, globally affecting to the different layers of the vein wall [9–11]. Therefore, it is known that a change in the normal homeostasis of the tissue conforming the vein wall occurs. Hereof, it has been described the potential determinant role of different axis such as the IGF-1/PAPP-A/STC-2 axis in tissue homeostasis [12].

Insulin-like growth factor-1 (IGF-1) is a growth factor implied in a great variety of metabolic processes of proliferation, growth, and cellular differentiation. Its levels are directly correlated to growth hormone (GH) levels, accomplishing all these effects through bounding to IGF type 1 receptor (IGF1R) [13]. Insulin and IGF-1 share a great structural similarity, even though they bound to different receptors; cellular signalisation mediated by both of them is almost identical, highlighting their interaction with some factors like Akt or FoxO [14].

The role of the pregnancy-associated plasma protein A (PAPP-A) has been described as a negative regulator of the levels of IGFBPs, especially of IGFBP 2, 4, and 5. PAPP-A indirectly controls the effects of IGF-1 from the sectioning of IGFBPs, potentiating their cellular effects by increasing their bioavailability [15]. The role of PAPP-A in different vascular conditions, such as atherosclerosis, has been demonstrated, having been associated with an increase of inflammation and endothelial dysfunction along with proliferation and migration of smooth muscular cells [16], processes that are as well important for the pathogenesis of varicose veins. In addition, PAPP-A is a protein which can be determined in serum, where its usage as a biomarker for several vascular pathologies has been demonstrated [17–19]. On the other hand, stanniocalcin 2 (STC-2) has been depicted as an inhibitor of the proteolytic activity of PAPP-A, having been demonstrated multiple alterations of the PAPP-A/Stc-2 axis in a great variety of conditions, such as diabetes mellitus, cancer, and cardiovascular disorders [12].

Due to all of these reasons, this study is aimed at analyzing the expression of the different components of this axis in the vein wall from CVeD patients in comparison to the control patients without venous disease (HV), using immunohistochemistry techniques (IHC) and real-time PCR (RT-qPCR).

## 2. Patients and Methods

### 2.1. Study Design

This is a cross-sectional study of 35 patients clinically diagnosed with CVeD (47,000 [27,000-68,000] years old) in comparison to 27 control patients without venous disease (HV) (45,000 [23,000-66,000] years old), *p* = 0.198. In the CVeD group, we had 22 women and 13 men, while in the HV group, we had 20 women and 7 men, *p* = 0.349. We observed that in CVeD patients, there were 22 patients with a family history of CVeD, while in HV, there were 18 patients, ^∗^*p* = 0.021.

The inclusion criteria were the following: women and men diagnosed with CVeD, with a body mass index (BMI) less than or equal to 25; with and without venous reflux in the greater saphenous vein; signed informed consent; and commitment to undergo follow-up during the pre- and postoperative periods, as well as to provide tissue samples. The exclusion criteria were patients with venous malformations or arterial insufficiency, patients without access to their clinical history, patients with pathologies that could affect the cardiovascular system (infectious diseases, diabetes, hypertension, and dyslipidemia), patients with toxic habits, and those uncertain of participating in the monitoring. The clinical diagnosis of CVeD and the evaluation of venous reflux were based on a noninvasive color Doppler ultrasound (7.5 to 10 MHz) of the superficial and deep vein systems. The saphenous vein segments for the HV group were verified during organ extraction for bypass surgery.

This study was conducted according to basic ethical principles (autonomy, harmless, benefit, and distributive justice); its development follows the standards of Good Clinical Practice and the principles enunciated in the last Declaration of Helsinki (2013) and the Oviedo Convention (1997). The project was approved by the ethics committee of the Gómez-Ulla Military Hospital (37/17).

### 2.2. Sample Processing

The totality of the mayor saphenous vein was extracted via saphenectomy. These fragments were introduced in two sterile tubes, one containing a RNA later solution (Ambion, Austin, TX, USA) and another containing MEM medium (Minimum Essential Medium) with 1% of antibiotic/antimycotic (Thermo Fisher Scientific, Waltham, MA, USA). Samples were processed in a Telstar AV 30/70 Müller class II 220 V 50 MHz laminar flow bell (Grupo Telstar SA, Terrassa, Spain) in a sterile environment. Samples collected in the RNAlater solution were kept at -80°C until been processed for the analysis of gene expression. Samples preserved in MEM were used to carry out histological studies. Samples were washed and hydrated multiple times with MEM without antibiotic to remove the erythrocytes and isolate the venous tissue. Subsequently, the venous tissue was sectioned in fragments and samples were preserved in F13 fixative (60% ethanol, 20% methanol, 7% polyethylene glycol, and 13% distilled water). After the time needed to fix it, samples were dehydrated in increasing concentrations of alcohol and were subjected to paraffin inclusion process. Once the tissue is impregnated, a paraffin block is confectioned. From these blocks, fine five-micron sections were made in a HM 350 s rotation microtome (Thermo Fisher Scientific, Waltham, MA, USA) and collected in slides impregnated with a 10% polylysine solution in order to accomplish the adherence of the sections to the slides.

### 2.3. Genetic Expression Study

The expression of the genes of interest was studied by real-time PCR method (RT-qPCR), in which the amount of cDNA was quantified in each of them. The RNA extraction was carried out by the guanidine-phenol-chloroform isothiocyanate method, following the procedure described by Ortega et al. [20]. Used primers were designed by the Primer-BLAST tool [21] and the AutoDimer application [22]. The StepOnePlus™ system was used with the relative standard curve method to carry out the quantitative PCR (qPCR). 5 *μ*L from each sample, previously diluted in nucleases free water, was mixed with 10 *μ*L of the intercalant agent IQ™ SYBR® Green Supermix (Bio-Rad Laboratories), 1 *μ*L of forward primer, 1 *μ*L of reverse primer, and 3 *μ*L of free DNases and RNases water in a MicroAmp® 96-well plate (Applied Biosystems-Life Technologies), obtaining a final volume of 20 *μ*L. Final results were normalized and compared to the GAPDH constitutive gene expression (Table 1). Data obtained from each gene were interpolated in a standard curve. In the 96-well plate, samples were triplicated and duplicated in a standard curve, and the two remaining wells were filled with negative controls.

### 2.4. Immunohistochemistry Studies

The antigen-antibody reaction was detected by the ABC method (avidin-biotin complex) with peroxidase or alkaline phosphatase as chromogen, in accordance with the protocol described by Ortega et al. [23]. The blocking of nonspecific bounding sites was carried out with 3% bovine serum albumin (BSA) and PBS overnight at 4°C (Table 2(a)). The incubation with the secondary antibody bounded to biotin was diluted in PBS for one and a half hour at room temperature (Table 2(b)).

An incubation with the ExtrAvidin®-Peroxidase avidin-peroxidase conjugate (Sigma-Aldrich, St. Louis, MO, USA) was carried out for 60 minutes at room temperature (diluted to 1 : 1200 on PBS). The incubation was revealed with the diaminobenzidine chromogenic substrate (DAB Kit, SK-4100) (Vector Laboratories, Burlingame, CA, USA). The chromogenic substrate was prepared immediately before exposition (5 mL of distilled water, 2 drops of tampon, 4 drops of DAB, and 2 drops of hydrogen peroxide). This technique allows a brown colored stain. In order to obtain contrast with the nuclei stain, incubation in Carazzi's haematoxylin was carried out for 5-15 minutes mount in aqueous medium with Plasdone. In every genetic study, sections from the same tissue were used as negative control, in which the incubation with the primary antibody was substituted with incubation with blocking solution.

### 2.5. Statistical Analysis in Interpretation of Results

For the purpose of the statistical analysis, the GraphPad Prism® 5.1 statistical program was used, and the Mann–Whitney *U* and *χ*^2^ Pearson's test were applied. The data are expressed as the median with interquartile range (IQR). Error bars on the figures are expressed with IQR. The significance values were set at ^∗^*p* < 0.05, ^∗∗^*p* < 0.005 , and ^∗∗∗^*p* < 0.001.

The preparations were examined under a Zeiss Axiophot optical microscope (Carl Zeiss, Oberkochen, Germany). Given the important role of the proteins involved, the assessment of the histological results was performed by the intensity of expression for immunohistochemical staining with a score of 1 to 3. Henceforth, histological samples from patients were classified as negative (0) or positive (1-3) IRS score method [24]. For each established group of subjects, 7 randomly selected microscopy fields were examined in each of the 5 sections. Subjects were classified as positive when the average proportion of the labelled sample was greater or equal to 5% of the total sample. This was done by calculating the total percentage of tissue marked in each microscopy field to obtain the value of mean for the study sample as described by Cristóbal et al. [25]. The observation and quantification of the samples were independently performed by two investigators.

## 3. Results

### 3.1. Increased Expression of IGF-1 in the Vein Wall of CVeD Patients

**Figure 1:**
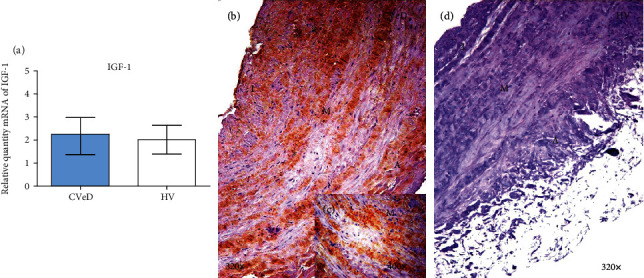
(a) Expression of mRNA for IGF-1 in CVeD (chronic venous disease) and HV (healthy controls) in arbitrary units. (b–d) Images showing immunostaining for IGF-1 in CVD patients in the three venous wall robes (asterisk) and CV. I: intimate robe; M: middle robe; A: adventitious robe.

Genetic expression study of IGF-1 showed no significant differences between the study groups, although it did show an upward trend in CVeD patients (CVeD = 2.250 [0.630 − 4.013] vs. HV = 2.014 [1.036 − 3.068], *p* = 0.5559) (Figure 1(a)).

In contrast, a significant increase in the IGF-1 protein expression was observed in the vein wall from CVeD patients (CVeD = 2.000 [0.500 − 3.000] vs. HV = 1.000 [0.000 − 2.250], ^∗^*p* = 0.0104). The microscopic analysis showed how the expression of IGF-1 was located in the three tunics of the vein wall (Figure 1(b)). An increased intensity was observed in the smooth muscular fibres and extracellular matrix of the vein wall from CVeD patients (Figure 1(c)).

### 3.2. CVeD Patients Show a Significant Increase of PAPP-A

**Figure 2:**
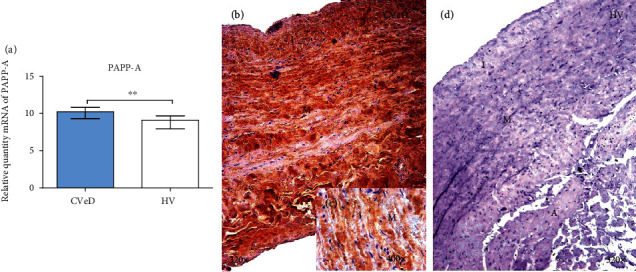
(a) Expression of mRNA for PAPP-A in CVeD (chronic venous disease) and HV (healthy controls) in arbitrary units. (b–d) Images showing immunostaining for PAPP-A in CVD patients in the three tunics of the venous wall (asterisk) and CV. I: intimate robe; M: middle robe; A: adventitious robe. ^∗∗^*p* < 0.005.

Genetic expression study of PAPP-A in the vein wall showed a significant increase in CVeD patients in comparison to HV (CVeD = 10.230 [7.846 − 11.726] vs.HV = 9.089 [7.324 − 10.013],  ^∗∗^*p* = 0.0029) (Figure 2(a)).

Protein expression of PAPP-A was significantly increased in the vein wall from CVeD patients (CVeD = 1.500 [0.250 − 3.000] vs.HV = 0.500 [0.000 − 1.500],  ^∗∗∗^*p* = 0.0002). PAPP-A was located in the three tunics of the vein wall from CVeD patients (Figure 2(b)). It is worth noting how the expression of PAPP-A was intensely located in the smooth muscular fibres of the vein wall from these patients (Figure 2(c)).

### 3.3. Significant Decrease in the Expression of STC-2 amongst CVeD Patients

**Figure 3:**
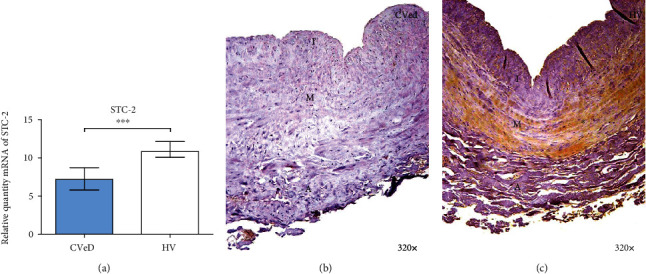
(a) Expression of mRNA for STC-2 in CVeD (chronic venous disease) and HV (healthy controls) in arbitrary units. (b, c) Images showing the immunostaining for STC-2 in CVD patients in the three tunics of the venous wall (asterisk) and CV. I: intimate robe; M: middle robe; A: adventitious robe. ^∗∗∗^*p* < 0.001.

A significant decrease in the genetic expression of STC-2 was observed amongst CVeD patients in comparison to HV by RT-qPCR (CVeD = 7.190 [3.296 − 11.324] vs.HV = 10.845 [9.079 − 13.389],  ^∗∗∗^*p* < 0.0001) (Figure 3(a)).

Protein expression study performed by immunohistochemistry techniques showed in a similar fashion a significant decrease (CVeD = 0.250 [0.000 − 1.000] vs.HV = 1.000 [0.000 − 2.500],  ^∗∗^*p* = 0.0056). The microscopic analysis showed SCT-2 was mainly located in the medium tunic of the vein wall from HV patients (Figure 3(c), asterisk).

## 4. Discussion

CVeD causes a severe alteration in the homeostasis of the vein wall, unchaining the activation of both cellular and systemic responses [11]. It has been found that this process is associated with different pathologic mechanisms which are key in the development of the disease such as an increase in the process of apoptosis and cellular death [26], deregulation in the synthesis of some cytokines [27], and even with some cell signaling pathways and changes in the extracellular matrix [28].

The impact that IGF-1 signaling can have on the aging process has long been known. Various animal models ranging from yeasts to human beings have demonstrated that lower levels of IGF-1 are associated with an increased longevity and a deceleration of the aging process [29]. However, it is known that the effects and levels of IGF-1 both at a physiologic level and a pathologic level depend on tissue, gender, and age of the subject [30], and it has also been demonstrated how the alteration of its signaling is associated with the appearance of several aging-associated diseases [31].

Previous studies have shown the existence of an increase in IGF-1 levels and its receptor, IGF-1R in varicose veins, in comparison to control veins, thus demarcating the important role it can play in the pathogenesis of the disease [32]. Moreover, the role of IGF-1 regarding the activation of the PI3K/Akt/mTOR pathway has been demonstrated and how these components were jointly involved in aging process, as well as its associated diseases [33]. Jia et al. [34] reported how IGF-1-mediated signaling promoted the phosphorylation of PI3K and Akt in smooth muscular cells of saphenous veins, promoting their proliferation *in vitro*. Along the same lines, our previous results showed that the PI3K/Akt/mTOR pathway was altered in CVeD patients, showing a direct association between greater involvement of this pathway and both premature and asynchronous aging of the venous tissue [28]. There are also more studies which show how IGF-1 promotes the activation of different signaling pathways, such as MAPK [35]. It has been demonstrated the existence of a greater activation of some components in this pathway, such as ERK1/2 in CVeD patients, especially in those who present venous reflux [36]. Our results show how the increase of IGF-1 could be associated with these statements, collaborating with the detriment suffered by venous tissue.

Amongst the different regulatory mechanisms of IGF-1, pregnancy-associated plasma protein A (PAPP-A) is one of the most studied elements vitally important in the regulation of this factor [37]. Although PAPP-A was initially found and identified in trophoblasts of the placenta from pregnant women, it has been demonstrated how it is also expressed in a vast range of cells and tissues, including fibroblasts and smooth muscular cells, both of great relevance in the pathogenesis of the varicose vein [7, 38].

PAPP-A is a protein which undergoes very strict regulation in the cell. On the one hand, it is known that its expression can be modulated by the synthesis of some proinflammatory cytokines, especially by IL-1*β* and TNF-*α* [37]. The role of inflammation in CVeDs has been extensively studied. It is known that the inflammatory process accompanies the development of the diseases as a consequence of the tissue damage associated with the situation of venous hypertension and valvular incompetence, observing an increase in leukocyte infiltration, as well as in the synthesis of proinflammatory cytokines [39]. It has been demonstrated that in atheromatous coronary arteries, this macrophage-mediated proinflammatory environment induces the expression and secretion of PAPP-A in both endothelial and smooth muscular cells [40]. In addition, as previously mentioned, PAPP-A increases the bioavailability of IGF-1 and the signaling mediated by its receptor in an autocrine and paracrine manner in the tissue [41], having been described the role of IGF-1 in the stimulation of the inflammatory response and the synthesis of cytokines [42].

Our results suggest that in the vein wall from CVeD patients, the inflammation associated with this condition could be acting similarly by increasing the expression of PAPP-A, in a positive feedback process. This fact is involved in promoting other main pathogenic processes, such as endothelial dysfunction [43].

On the other hand, there are different mechanisms which negatively regulate PAPP-A levels in the cell, those that are carried out by a series of proteins depicted as stanniocalcins 1 and 2 [44]. Interestingly, it is known that Stc-2, contrarily to Stc-1, covalently bounds to PAPP-A, performing both essential functions in the regulation of calcium levels and cellular response to different conditions as oxidative stress or endoplasmic reticulum stress, hence being fundamental in cellular homeostasis [45, 46]. We previously demonstrated the existence of an increase in oxidative stress damage markers and in the lipidic peroxidation process in CVeD patients [11]. Our results show how decreasing Stc-2 levels could support this fact, in addition to implying the loss of an inhibition mechanism of the proteolytic activity of PAPP-A and, therefore, of the signaling regulation mediated by IGF-1 in the venous tissue that appears in CVeD patients.

The analysis of the serum levels of these components and their possible use as biomarkers is also currently in progress. It is important to note that circulating PAPP-A can be found in 2 different forms: a first form associated with pregnancy, covalently bound to the proteolytically inactive eosinophil major basic protein (proMPB), and a second form produced by vascular cells that are not covalently bound to proMBP and that do exhibit proteolytic activity, which this distinction is being important for its measurement and interpretation under different conditions [47]. The presence of Stc-2 in serum has also been identified, as well as IGFBP-4, which is regulated by PAPP-A [48]. Although PAPP-A also modulates other IGFBPs, such as IGFBP 2 and 5, IGFBP-4 exclusively appears to be regulated by the proteolytic activity of PAPP-A. Taking into account all of these reasons, the Stc-2/PAPP-A/IGF-1 axis is necessary to know all the physiopathologic mechanisms involved in vascular diseases [49–54].

## 5. Conclusions

Our study is pioneering in showing how CVeD patients have a significant increase of PAPP-A expression in their vein wall. These markers may be quite relevant as CVeD markers. Further studies should be oriented to the measurement and assessment of different components of this axis which may serve as serum biomarkers in venous disease patients. However, our study is the first to highlight the presence and importance of IGF-1/PAPP-A/STC-2 markers in CVeD.

## Figures and Tables

**Table 1:** Primer sequences used in RT-qPCR and temperature (Tm).

Gene	Sec. Fwd (5′ → 3′)	Sec. Rev (5′ → 3′)	Tm
GAPDH	GGAAGGTGAAGGTCGGAGTCA	GTCATTGATGGCAACAATATCCACT	60
IGF-1	GCTCTTCAGTTCGTGTGTGG	CGCAATACATCTCCAGCCTC	69
STC-2	GCTCTCGGTCCCGTCAC	GACTCAGGAGAGCTCGACAC	51
PAPP-A	CCCAGGCAGTCAGATCATCTTC	AGCTGCCCCTCAGCTTGA	52

**(a):** 

Antigen	Species	Dilution	Provider	Protocol specifications
IGF-1	Rabbit	1 : 100	Abcam (ab263903)	10 mM sodium citrate, pH = 6 before incubation with blocking solution
PAPP-A	Mouse	1 : 500	Abcam (ab52030)	0.1% Triton in PBS, 10 minutes before incubation with blocking solution
STC-2	Rabbit	1 : 300	Abcam (ab261915)	—

**(b):** 

Antigen	Species	Dilution	Provider	Protocol specifications
IgG (mouse)	Goat	1 : 300	Sigma (F2012/045k6072)	—
IgG (rabbit)	Mouse	1 : 1000	Sigma (RG-96/B5283)	—

## Data Availability

The data used to support the findings of the present study are available from the corresponding author upon request.
